# Ultrasound: Basic understanding and learning the language

**DOI:** 10.4103/0973-6042.76960

**Published:** 2010

**Authors:** Barys Ihnatsenka, André Pierre Boezaart

**Affiliations:** 1 Department of Anesthesiology, Division of Acute Pain Medicine and Regional Anesthesia; 2 Department of Orthopedic Surgery and Rehabilitation, University of Florida College of Medicine, Gainesville, Florida, USA

**Keywords:** Hyperechoic, hypoechoic, anechoic, musculoskeletal, ultrasound, regional anesthesia, ultrasound knobology, ultrasound probe selection and manipulation

## Abstract

Ultrasound (US) use has rapidly entered the field of acute pain medicine and regional anesthesia and interventional pain medicine over the last decade, and it may even become the standard of practice. The advantages of US guidance over conventional techniques include the ability to both view the targeted structure and visualize, in real time, the distribution of the injected medication, and the capacity to control its distribution by readjusting the needle position, if needed. US guidance should plausibly improve the success rate of the procedures, their safety and speed. This article provides basic information on musculoskeletal US techniques, with an emphasis on the principles and practical aspects. We stress that for the best use of US, one should venture beyond the “pattern recognition” mode to the more advanced systematic approach and use US as a tool to visualize structures beyond the skin (sonoanatomy mode). We discuss the sonographic appearance of different tissues, introduce the reader to commonly used US-related terminology, cover basic machine “knobology” and fundamentals of US probe selection and manipulation. At the end, we discuss US-guided needle advancement. We only briefly touch on topics dealing with physics, artifacts, or sonopathology, which are available elsewhere in the medical literature.

## INTRODUCTION

Ultrasound (US) use has rapidly entered the field of acute pain medicine and regional anesthesia and interventional pain medicine over the last decade, and it may even become the standard of practice.[1] US guidance for nerve blocks and interventional pain management techniques may have several potential advantages over conventional landmark-based techniques that assume minimal anatomical variation between persons, or nerve stimulation-assisted techniques that are based on the premise that an appropriate motor response is the perfect surrogate marker for needle proximity to the sensory fibers of a nerve. These assumptions, of course, are not entirely correct, and could potentially be responsible for block failure or block placement difficulties when these conventional techniques are used. The advantages of US guidance include the ability to both view the targeted structure and visualize, in real time, the distribution of the medication throughout and relative to the tissue (e.g., nerve tissue), as well as the capacity to control its distribution by readjusting the needle position, capabilities which should plausibly improve the success rate of the procedures. The ability to visualize the targeted structure and other structures of importance, such as blood vessels, lung, or other organs, should, logically, also improve the speed and safety of the procedures.[2]

Compared to the use of fluoroscopy-guided procedures that can only visualize bony tissue, US additionally allows the visualization of soft tissues. US equipment is also more portable and less expensive. Moreover, even regular use of US does not place patients and practitioners at risk of harmful radiation exposure, although this may be a matter of debate.[3]

It should be clearly understood from the outset that the ability to “see” the targeted structure with US does not preclude a thorough knowledge of gross or micro-anatomy. Some experts agree that proper utilization of US requires an even better knowledge of applied anatomy than that required for conventional techniques of nerve localization. For the best use of US in acute pain medicine and regional anesthesia, one should venture beyond the “pattern recognition” mode to the more advanced systematic approach and use US as a tool to visualize structures beyond the skin (advanced sonoanatomy mode). ‘Pattern recognition’ refers to memorization of an US image of a targeted structure (textbook picture) and learning the maneuvers and techniques necessary to acquire the image. This is, however, not sufficient in the presence of anatomical variations or if US is used for diagnostic purposes. It also implies the need for the continuous presence of a teacher to confirm the image obtained and the required maneuver for different block variations (personal observation). To advance beyond the pattern recognition mode, a thorough knowledge of applied anatomy combined with a basic understanding of how a 2-dimensional (2-D) US image represents a 3-dimensional (3-D) anatomical structure is needed; the latter is the goal we wish to accomplish in this paper for the readership.

This article will provide basic information on musculoskeletal US techniques, with an emphasis on the principles and practical aspects. We discuss the sonographic appearance of different tissues, introduce the reader to commonly used US related terminology, cover basic machine “knobology” and fundamentals of manipulation of the US probe and US-guided needle advancement. We will only briefly touch on topics dealing with physics, artifacts, or sonopathology, which are available elsewhere in the medical literature.[4–6] We hope to, in the future, publish an article that will provide an exhaustive review concerning the use of sonoanatomy of the neck above the clavicle.

US images in this paper were taken with a straight array 38-mm, high frequency probe (6–13 MHz), although one image was taken with a curved array 60-mm, low frequency probe (2–5 MHz) on an S-nerve US machine (S-Nerve, Sonosite, Bothell, WA, USA). Use of other equipments, especially curved probes, which have smaller “footprints,” will produce different images.

## IDENTIFYING DIFFERENT TISSUE TYPES AND UNDERSTANDING ULTRASOUND TERMINOLOGY

### Echogenicity

**Figure 1:**
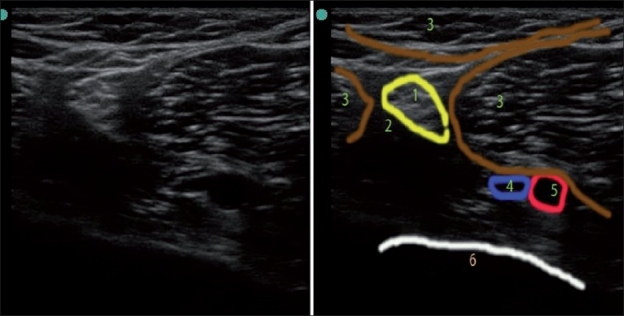
US image of popliteal area. 1) Sciatic nerve (hyperechoic with stippled “honeycomb” structure); 2) Adipose tissue (hypoechoic); 3) Muscles (note the striations and hyperechoic fascial lines on muscle surfaces); 4) Vein (anechoic – partially collapsed under pressure to US transducer); 5) Popliteal artery (anechoic – pulsating); 6) Bone (hyperechoic rim with hypoechoic shadow below it)

Echogenicity of the tissue refers to the ability to reflect or transmit US waves in the context of surrounding tissues.[7–9] Whenever there is an interface of structures with different echogenicities, a visible difference in contrast will be apparent on the screen. Based on echogenicity, a structure can be characterized as hyperechoic (white on the screen), hypoechoic (gray on the screen) and anechoic (black on the screen) [Figure 1].

**Figure 2:**
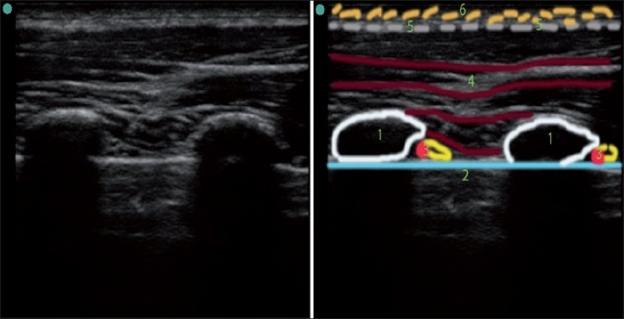
US image of thorax (ribs in short axis). 1) Rib (short-axis view, note the hyperechoic rim and intense acoustic shadow below it) 2) Pleura with lung below (pleural sliding and shimmering, as well as comet tails artifact is seen only during live scan) 3) Neurovascular bundle 4) Muscles 5) Fascia 6) Adipose tissue

*Bone* appears black or anechoic on US, with a bright hyperechoic rim [Figures 1 and 2]. Because the US beam cannot penetrate bone, it casts an acoustic shadow beyond it. Cartilage appears hypoechoic, and is more penetrable by US than bone. Blood vessels also appear black or anechoic [Figure 1]. Veins are usually easily collapsible upon external pressure by the transducer, while arteries are pulsatile and do not collapse with moderate pressure. Blood vessels have a distinct appearance on color Doppler mode: flow toward the probe appears red, while flow away from the probe appears blue. A useful mnemonic used by radiologists is BART, i.e., Blue Away, Red Toward. Muscles are hypoechoic with striate structure; fat is almost anechoic, while *fascia* and other connective tissue strands and fascicles appear as hyperechoic lines [Figures 1 and 2]. Lymph nodes appear anechoic or hypoechoic. The appearance of nerves is variable, depending on the proximity to the neuraxium. Proximal nerves are hypo-anechoic (approximately similar to blood vessels but neither collapsible nor pulsatile), and distal nerves are hyperechoic, with a stippled (“honeycomb”) structure (with hypo-anechoic fascicles on the hyperechoic background of connective tissue surrounding them) [Figure 1]. Ligaments and tendons have a similar appearance to distal nerves (hyperechoic, but not “honeycomb”). If in doubt, one can trace the “target structure” proximally or distally in order to distinguish the nerve from a tendon based on anatomy (the tendon will be traceable to the muscle body). Tendons have characteristic striation in the long-axis view, and are more *anisotropic* (discussed later) than nerves. The *lung* has a very distinct appearance [Figure 2]; one can usually visualize a “shimmering”, hyperechoic pleura sliding in rhythm with each breath, as well as *comet tail* artifacts, if US is performed while the patient is breathing; these are images that cannot be appreciated on static pictures. Loss of sliding and shimmering pleura and comet tail artifact may be due to pneumothorax.[7–9]

### Scanning planes

Scanning planes are similar to the well-known anatomical planes: axial (transverse), sagittal, parasagittal, and coronal.[10] “Oblique” direction can be combined with any standard plane to create, for example, a “parasagittal oblique” or “transverse oblique” scanning plane.

### Ultrasound views

All subjects, except cubes and spheres (that are absolutely symmetrical in all directions), have a long axis and a short axis when viewing them from a 2-D approach. Viewing a structure in the long axis will provide a long-axis view, and vice versa; an oblique view is also possible.

Anatomical structures, such as vessels or nerves, are more commonly viewed in the short axis (round shape on the screen) than long axis when the operator loses the lateral–medial perspective. Rotating an US probe to 90° will change a short-axis view into a long-axis view, and vice versa. An oblique view can be appreciated during rotation of the probe between the true short axis view and the long-axis view.

### Angle of incidence

**Figure 3:**
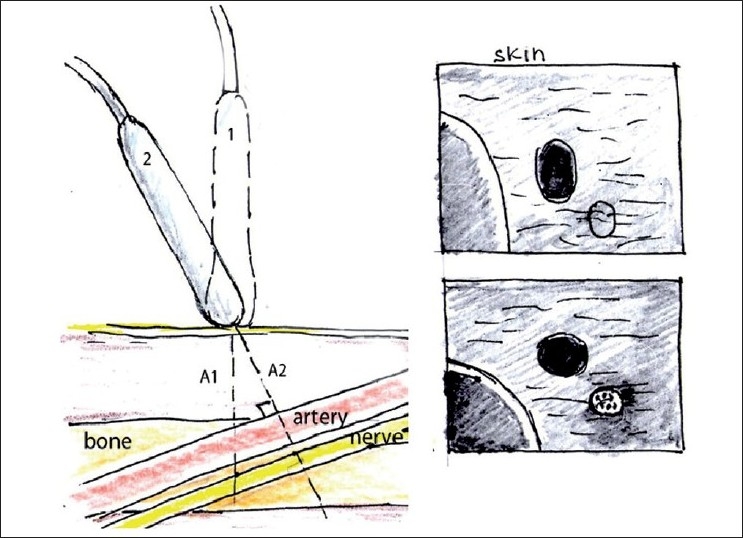
Schematic illustration of improving the angle of incidence by tilting the probe. By tilting the probe from position 1 to position 2, we obtained the true axial short-axis view of the artery and the nerve. The shape of the image of the artery and the nerve got more rounded, and the image of the nerve is much more defined in position 2 due to a more favorable angle of incidence. Note the changes in A1 and A2 distance as well

The angle at which the US waves encounter the surface of the structure, termed, the angle of incidence, affects the way it is presented on the screen. If the angle is perpendicular, or close to perpendicular, more US waves will be reflected back to the transducer and fewer will be “scattered” away, resulting in a better image. If the US waves are more parallel to the surface of the object (more than a 45° angle of incidence), the image will have less definition. The operator can improve the image of the target by tilting or rotating the probe, thus adjusting the angle of incidence [Figure 3].

**Figure 4:**
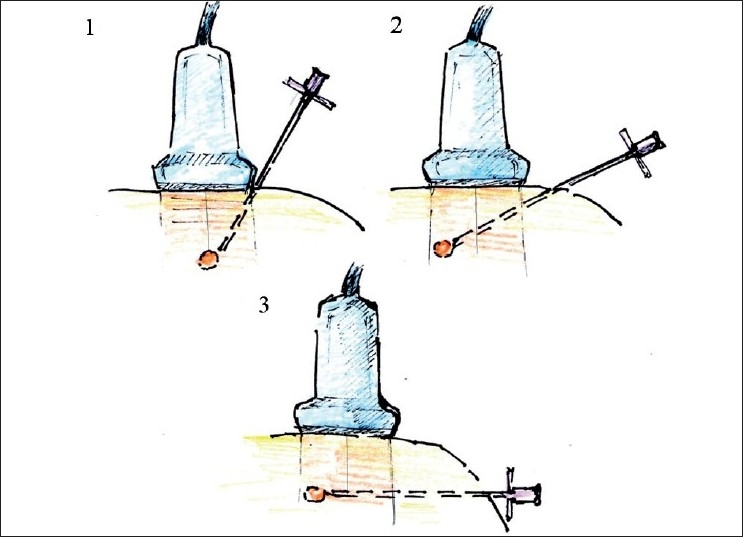
Improving needle visualization during “in plane” needle placement. To improve needle visualization, one can change the US probe position (from 1 to 2) and the needle approach (from 1 to 2 to 3) to optimize the angle of incidence between US waves and the needle

A close-to-perpendicular angle of incidence is also very important for better needle visualization during US-guided needle insertion, and can be achieved by changing the needle approach such that it is advanced more perpendicular to the US waves [Figure 4].

### Anisotropy

Anisotropy in ultrasonography could be defined as a tissue property that is responsible for changes in the US reflection dramatically, even with mild changes in the angle of incidence. It creates the phenomenon known as “now-you-see-me-now-you-don’t”. Different tissues have varying degrees of anisotropy. Nerves and tendons are notoriously anisotropic and could make US-guided nerve blocks quite challenging. Tendons are slightly more anisotropic than peripheral nerves, a factor that occasionally can be used for differentiating structures that may look similar on US, although tracing the structures more proximally or distally to verify anatomical relationship is still a better way of doing it. US probe maneuvers, such as pressure, tilt, and rotation, are primarily performed to optimize the angle of incidence in order to get the best reflection of the targeted structure.

## ULTRASOUND WAVE FREQUENCY, IMAGE RESOLUTION, AND PENETRATION

High frequency probes (10–15 MHz) and midrange frequency probes (5–10 MHz) provide better resolution but have less penetration. High frequency probes are, therefore, preferred for US imaging of superficial structures (2–4 cm), while midrange frequency probes are preferred for slightly deeper structures (5–6 cm). However, when US imaging of deep structures (for example, a proximal sciatic nerve that can be as much as 10 cm deep) is required, a low frequency probe (2–5 MHz) is preferred, although the quality of the image will be substantially poorer. When determining the correct choice between probes with different US frequencies, choose the one that will provide the best resolution for the required depth. Most practitioners have several different probes for more flexibility.

## CURVILINEAR VERSUS STRAIGHT PROBE

**Figure 5:**
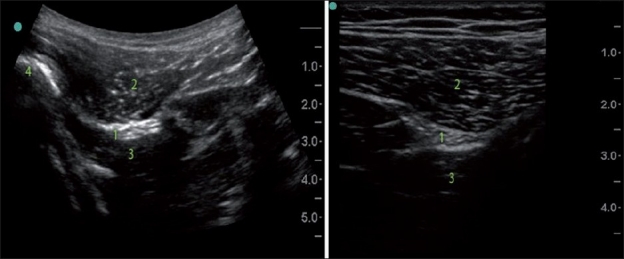
Curved low frequency US probe image versus straight high frequency probe image (subgluteal sciatic nerve). Note the difference in the shape and the scope of the images, different depth and resolution. 1) Sciatic nerve; 2) Gluteus maximus; 3) Quadrates femoris; 4) Femur

Curvilinear probes generate a wedge-shaped US beam and a corresponding image on the screen[8] [Figure 5, left image]. The curved image of the anatomical structures which, in reality, are straight may initially look peculiar but, with time, the operator becomes accustomed to the view. The curved probe can easily roll on its scanning surface, thus affecting the direction of the US beam. This ability to roll the probe is occasionally advantageous in allowing us to “look around the corner”, but it can also have a disadvantage in that extra efforts are needed to keep the US beam perpendicular to the skin surface while looking straight down.

The curvilinear probe provides a broader view that could be obtained via a smaller acoustic window; the image of deeper structures is wider than the footprint of the probe. This factor of widening of the image with the depth should be also considered during distance measurement. In general, determining the precise depth of the structure and width assessment with a curved probe is tricky. It is necessary to understand that the width of the image is equal to the probe footprint size only at the uppermost part of the image, and the depth marks on the side of the screen are pertinent only for measurement of the depth on the line drawn through the middle of the probe.

The curvilinear probe may be superior to the straight probe in its ability to visualize the needle that advanced in plane at the steep angle because it provides a more favorable angle of incidence.

Straight probes produce a straight US beam and an image with a width equal to the size of the transducer footprint from the surface to the deeper structures [Figure 5, right image].

## ULTRASOUND PROBE FOOTPRINT SIZE

The smaller footprint probe may be advantageous when negotiating the small anatomical convexity and concavity of the body surface, and may provide better contact between the probe and skin, which is especially useful while using US in uneven areas (supraclavicular or infraclavicular, for example), especially for children. When the footprint of the probe is too small, vision becomes “tunneled”, although a larger footprint will not only give a “wider picture” but also improve lateral resolution. A small footprint of the probe is occasionally more advantageous for in-plane needle advancement, allowing the operator to place the needle entry closer to the target and thus also shortening the distance to the target.

## COLOR DOPPLER FUNCTION

**Figure 6:**
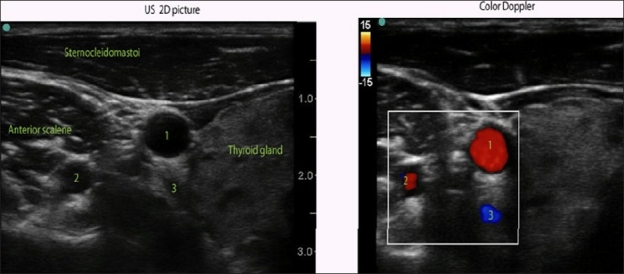
Color Doppler over 2-D US of the anterolateral neck area at C6/C7 level. While looking only at the 2-D US image, one may confuse vertebral artery (2) with a nerve root. Both may look the same on 2-D US. Color Doppler helps to distinguish nerve roots from blood vessels. 1) Carotid artery; 2) Vertebral artery; 3) Inferior thyroid vein

Color Doppler helps to distinguish structures with movement, for example, blood moving within vessels. Because proximal nerves are usually hypo-anechoic and can be confused with blood vessels, this function may be especially helpful [Figure 6]. Color Doppler can also be used to determine the direction of the blood flow when needed.

Compared to 2-D US, Doppler works best when US waves are almost parallel to the direction of the moving object (that is, blood, as in the case of blood vessels). When the angle of incidence is close to 90° and flow is low, there may be no color on the screen, possibly producing a false negative indication of “no flow”.[5] To increase the sensitivity of vessel recognition, the probe should be tilted out, off the perpendicular angle of incidence. Power Doppler should also be used in these situations because it is more sensitive in recognizing low flow in small blood vessels despite an unfavorable angle of incidence than regular Color Doppler.[5]

## KNOBOLOGY : GAIN, FOCUS, AND MODE OF SCANNING

Changing the gain will change the amount of white, black, and gray on the monitor. Adjusting the gain of the image may improve the operator’s ability to distinguish structures on the screen; the amount of gain to use depends on personal preference. Most US machines have an auto-gain knob, which is commonly used.

Modern US machines have useful “nerve”, “angio” or “general” modes. The “focus” function available on these machines may help to improve visualization of the targeted structure, although it is rarely needed for superficial structures if the depth is set correctly.

## DEPTH SETTINGS

It is wise to begin with a somewhat higher depth setting in order to first get a “big picture”, and then gradually decrease the depth when the targeted structure is found. For US-guided injection, the depth should be set about 1 cm deeper than the target of interest. If another structure of importance, such as a vessel or lung, is situated below the target, the depth should be adjusted accordingly to produce a good view of the field and improve safety.

By knowing the target depth and its position on the screen, the initial angle of needle advancement can be estimated even before visualizing the needle on the screen. (If one uses a 4-cm-wide transducer and an in-plane approach, the initial angle will be close to 45° if the targeted structure is situated in the middle of the screen at the 2-cm depth and the needle is inserted at the edge of the transducer.) This angle should be adjusted as soon as the needle can be viewed on the screen and its trajectory is clear.

## PROBE ORIENTATION

Probe orientation is important because the US probe can be easily rotated around (180°) while the position of the monitor remains unchanged, which may create confusion in the direction of probe manipulation and needle advancement and placement. Therefore, it is always useful to confirm which side of the probe corresponds to a particular side of the screen in order to identify the correct orientation of the image. All transducers have an orientation marker that corresponds to the marker on the screen.

## PROBE MANIPULATION

**Figure 7:**
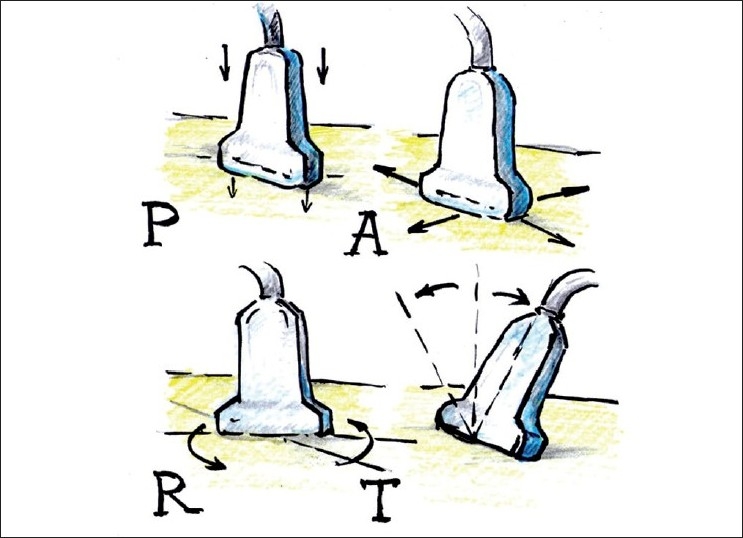
US probe manipulation maneuvers “PART” mnemonic for Pressure, Alignment, Rotation and Tilt as fundamental probe manipulation maneuvers

When dealing with US probe manipulation, the mnemonic PART (Pressure, Alignment, Rotation and Tilt) is useful [Figure 7]. It is important to understand that by manipulating the US probe, we primarily manipulate the direction of the beam, and, by changing the direction of the beam, slightly different US images of the same structures can be obtained.

### Pressure

Correct pressure application can considerably improve the image quality. It affects the echogenicity of the tissue and shortens the distance to the structure of interest. Ordinarily, pressure must be applied evenly to get the correct direction of the scan; however, occasionally, the operator may intentionally need to apply more pressure on one side of the probe in order to direct the US beam in the desired manner (angling the probe). Pressure to the probe is also applied to compress a vein or to push an anatomical structure out of the way of an intended needle pass. Excessive pressure, however, can cause discomfort to the patient. Placing excessive pressure on the transducer may also be responsible for significant depth underestimation to the relatively deep structure if US is used solely for marking and measuring, and if a procedure is done after that without US on a patient who has significant amount of soft tissue, which “springs back” when the probe is removed.

### Alignment (sliding)

The main goal of this maneuver is to find the structure of interest and position it optimally on the screen for needle advancement (usually in the middle of the screen for an out-of-plane approach and somewhat on the opposite side of the screen for an in-plane approach). Sliding is also very useful for tracing the potential structure proximally and distally for better verification of pertinent anatomy during a “scout scan”.

### Rotation

With rotation, one can achieve several goals. First, one can attain a true axial view of the target with its long axis parallel to the surface but not perpendicular to the current US plane. For example, if you image a blood vessel (that is parallel to the surface) in the short-axis view and slide a US probe along the vessel’s long axis, you must slightly rotate the probe when the vessel makes a turn in order to maintain true short axis view. Second, one can align the target into a more favorable trajectory for a safe needle pass (away from vessels or pleura, for example).

Rotation will affect the image if it brings the object out of the true axial view. If the long axis of the object remains parallel to the surface and the US probe is gradually rotated relative to the long axis of the structure, the round cross section of the true axial cut (of the normally round vessel or nerve, for example) will be replaced by a more oval shape. By continuous rotation of the probe of 90° from the initial probe position, one can change the view of the structure from its short axis to its long axis, and vice versa.

### Tilt

There is no particular recognized terminology to define the direction of the tilt, and confusion can arise from the fact that when the probe is tilted in one direction, the US plane, in fact, sweeps to the opposite direction.

**Figure 8:**
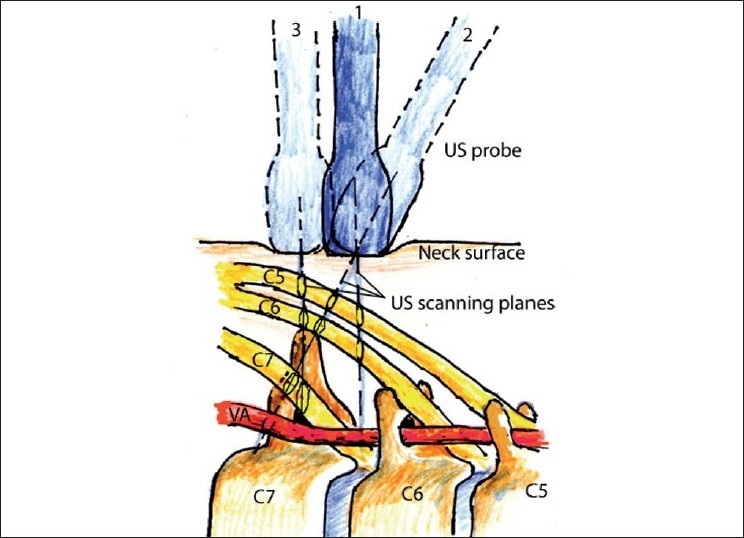
Schematic illustration of tilting and sliding during image acquisition in the neck. Position 1: The probe is perpendicular to the skin at the level between C6 and C7. Tilting the probe (position 2) allows us to see C7 transverse process and C7 nerve root. Sliding the probe more caudad (position 3) allows us to see an image similar to the image obtained from position 2 but with the probe perpendicular to the skin

Several goals can be achieved by tilting the probe. First, by sweeping the US beam in the particular direction desired by tilting the probe, one can “preview” the image by sliding the probe in the opposite direction of the tilt [Figure 8]. Second, by tilting the probe, a true short-axis view of the object can be obtained, the long axis of which is not perpendicular to the initial US beam plane [Figure 3, position 2].

As with rotation, if the US plane cuts the long axis of the target at the angle that is not perpendicular, it will distort the 2-D image [Figure 3, position 1]. By tilting the probe out of the true axial view of the target, the image will change as follows:

- The distance from the surface to the target will increase and
- The shape of the target on the screen will be untrue (oval instead of round, for example).

## ULTRASOUND ARTIFACTS

US artifacts are responsible for untrue images when we see on the monitor something that does not exist in reality or we do not see something that is in fact true. Many artifacts from US have been described; some are well understood and related to the physics of US, such as reverberation, mirror image, or acoustic enhancement artifacts, while others are not fully understood. These are outside of the scope of our paper; if detailed information is desired on these topics, the reader is referred to specialized texts.[6]

## SONOPATHOLOGY

A general description of sonopathology can be found in other articles.[11] As a rule of thumb, factors that negatively affect the echogenicity of tissues include the accumultion of extra water in soft tissue, as occurs with edema; loss of muscle mass, as happens with hypotrophy; and accumulation of micro-droplets of fat in the muscle, thereby producing an US image with less sharpness and contrast. Fluid collections could be readily seen by US and this has been used by radiologists for years. Air bubbles in the soft tissue can significantly affect the image to the point that it renders US unproductive. It stands to reason that a great deal of experience in imaging of normal sonoanatomy is needed before an operator can reliably visualize any pathology. Pure anatomical variations, such as unusual location of the nerve or presence of additional nerve or vessel, for example, are not considered pathological, provided these variations are not affecting normal function. Some anatomical variations that are missed on the exam before the nerve block, nevertheless, could be responsible for block failure.

## NEEDLE ADVANCEMENT TERMINOLOGY AND TECHNIQUES

**Figure 9:**
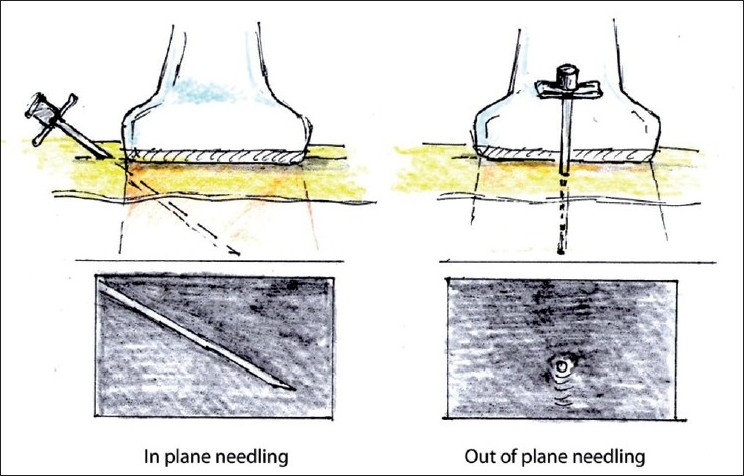
In- and out-of-plane needle placement

In-plane needle placement occurs when the needle can be seen on the US monitor in the long-axis view (long axis of the needle is situated within the US scanning plane). Out-of-plane needle placement occurs when the long axis of the needle is directed across the scanning plane so the needle can be seen in the short-axis view [Figure 9]. Although other approaches can be employed, in-plane needling is commonly used for single injections, while out-of-plane is used for catheter placement.

**Figure 10:**
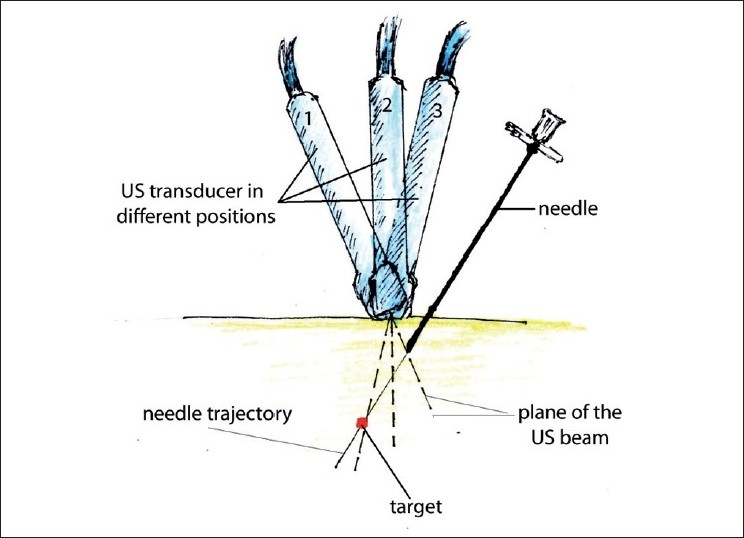
Improving tip of the needle visualization during out-of-plane needle placement. Gradual tilting of the probe during needle advancement allows the operator to follow the tip of the needle. Sliding a probe without tilting could be an alternative way of keeping the needle tip in the view

When performing out-of-plane needle advancement, dynamic tilting or sliding of the transducer when advancing the needle may help track the tip of the needle [Figure 10]. Visualizing the tip of the needle can be challenging, yet is essential. For this purpose, it is common to use tissue movement, or injections of small volumes of dextrose (if nerve stimulation is planned) or normal saline as an indicator (hydrolocation). Use of US, in combination with nerve stimulation, may be especially beneficial for the out-of-plane approach in some instances.

The in-plane mode is usually the preferred approach because it allows one to visualize the entire needle, including its tip. Visualization can be enhanced if a larger or specially designed echogenic needle is used and a more favorable angle of incidence is employed.[12]

## ERGONOMICS OF ULTRASOUND-GUIDED PROCEDURES

An US machine should be positioned on the contralateral side of the patient, with the operator standing on the ipsilateral side that needs to be blocked or examined. The transducer is usually held in the operator’s nondominant hand, with the needle in the dominant hand. The transducer should gently be held quite low on the probe, close to the scanning surface, rather than harshly gripped on the top of the handle. When planning to use the in-plane approach, it is preferable to place the probe directly perpendicular to the skin. (If it is possible to do without sacrificing image quality, try not to tilt the probe, as this will add difficulty with in-plane needle advancement; personal observation).

## PROBE MANIPULATION DURING NEEDLE ADVANCEMENT

It is important to stabilize the US probe position after obtaining the desired image. This can be facilitated by gently bracing the hand holding the probe on the patient’s body. Subsequently, we recommend taking the operator’s attention off the screen and focusing on attaining correct needle alignment with the US probe. Only after some advancement of the needle through the skin has occurred should the probe operator shift his/her attention back to the screen. At that point, if the needle can be visualized on the screen, further advancement and trajectory changes can be made based on feedback from the US screen without averting attention to the probe position and needle. Eye-hand coordination is required for this maneuver, and phantom exercises are very helpful to enhance this particular skill.[13]

Operators, particularly at the beginning of training, commonly lose sight of the tip of the needle or the entire needle from view. In these instances, it is perfectly proper to look back at the probe and find the best possible way to realign the needle with the US plane. If only the tip of the needle is “moving out” of view, one can slightly withdraw the needle back and try again with slight trajectory adjustment. Visualization of the needle can also be regained by slight probe manipulations (tilt, rotation, or sliding). It is important to develop a “feel of depth” for needle advancement and have accurate expectations for corresponding changes on the US screen should they occur. The US beam is very thin (about 1 mm wide), so even subtle movements can bring the needle in and out of the viewing field. If the operator does not have this “feel of depth” and corresponding expectation for the tip of the needle position on the screen, and tip of the needle accidently moves out of the US plane, the operator may advance the needle deep, before realizing it.

## SUMMARY

Knowledge of the fundamentals of US that were mentioned in this article, combined with understanding of the clinical 3-D anatomy of the area of the interest, will allow practitioners to use musculoskeletal US and US-guided procedures effectively and safely. We hope to publish an article dedicated to sonoanatomy of the neck above the clavicle in the near future.

## References

[1] Marhofer P, Chan VW (2007). Ultrasound-guided regional anesthesia: current concepts and future trends. Anesth Analg.

[2] Neal JM, Brull R, Chan VW, Grant SA, Horn JL, Liu SS (2010). The ASRA evidence-based medicine assessment of ultrasound-guided regional anesthesia and pain medicine. Executive summary. Reg Anesth Pain Med.

[3] Cory PC (2009). Concerns regarding ultrasound-guided regional anesthesia. Anesthesiology.

[4] Brull R, Macfarlane AJ, Tse CC (2010). Practical knobology for ultrasound-guided regional anesthesia. Reg Anesth Pain Med.

[5] Merritt CR, Rumack CM, Wilson SR, Charboneau JA (2005). Physics of ultrasound. Diagnostic Ultrasound.

[6] Sites BD, Brull R, Chan VW, Spence BC, Gallagher J, Beach ML (2007). Artifacts and pitfall errors associated with ultrasound-guided regional anesthesia. Part II: a pictorial approach to understanding and avoidance. Reg Anesth Pain Med.

[7] Bigeleisen PE (2010). Ultrasound-guided Regional Anesthesia and Pain Medicine.

[8] Pollard BA, Chan VW (2009). Introductory Curriculum for Ultrasound-Guided Regional Anesthesia.

[9] Tsui BC (2007). Atlas of Ultrasound and Nerve Stimulation-Guided Regional Anesthesia.

[10] Williams PL (1995). Gray’s Anatomy.

[11] Brian DS, Macfarlane AJ, Sites VR, Chan VW, Brull R (2010). Clinical sonopathology for the regional anesthesiologist. Reg Anesth Pain Med.

[12] Maecken T, Zenz M, Grau T (2007). Ultrasound characteristics of needles for regional anesthesia. Reg Anesth Pain Med.

[13] Pollard BA (2008). New model for learning ultrasound-guided needle to target localization. Reg Anesth Pain Med.

